# Optimized gating and reference ranges of reticulated platelets in dogs for the Sysmex XT-2000iV

**DOI:** 10.1186/s12917-016-0779-4

**Published:** 2016-07-22

**Authors:** Dana E. Oellers, Natali Bauer, Melanie Ginder, Sigrid Johannes, Iris Pernecker, Andreas Moritz

**Affiliations:** Department of Veterinary Clinical Sciences, Clinical Pathophysiology and Clinical Pathology, Justus-Liebig University Giessen, Frankfurter Str. 126, 35392 Giessen, Germany; IDEXX BioResearch Europe, A Division of IDEXX Laboratories, Ludwigsburg, Germany; Merck Serono, Global Non-Clinical Safety, Merck KGaA, Darmstadt, Germany

**Keywords:** RNA-rich platelets, Canine, Hematology analyzer

## Abstract

**Background:**

Canine reticulated platelets (r-PLTs) i.e., juvenile PLTs reflecting thrombopoiesis can be measured automatically with the hematology analyzer Sysmex XT-2000iV using manual gating options. However, the impact of interferences on r-PLT measurements performed with the gates published previously (Pankraz et al., Vet Clin Path 38:30–38, 2009; Gelain et al., High fluorescent platelets fraction in macrothrombocytopenic Norfolk terrier, 2010) is largely unknown.

The aim was to compare different published gates for measurement of r-PLTs with the Sysmex XT-2000iV with an own, optimized gate (“Oellers-gate”) and to establish reference intervals (RIs) in > 120 dogs.

Data of 362 measurements of diseased and healthy dogs were analyzed retrospectively. Several gates were applied and RIs for r-PLTs and platelet indices were established for pet dogs and a group of 153 healthy Beagles kept under defined housing conditions. Intra-assay precision (CV) was also assessed.

**Results:**

In 30/362 samples, interferences consistent with small erythrocytes/reticulocytes were seen in the previously published gates but not in the “Oellers-gate”. Good correlation was found between the different gates (r_s_: 0.88–1.00). RIs for the “Pankraz-gate”, the “Gelain-gate”, and the “Oellers-gate” were 0.0–1.2, 0.2–3.7 and 0.2–3.9 % respectively. CVs were ranging between 22 and 41 %.

**Conclusions:**

Optimization of previously published gates minimized interferences of small erythrocytes with r-PLT measurements.

## Background

Canine reticulated platelets (r-PLTs) i.e., juvenile platelets were first described in 1969 following acute blood loss in dogs [1]. Later, r-PLTs were detected with flow cytometry using Thiazol Orange [2] comparable to the measurement of reticulocytes [3]. Thiazol Orange positive platelets are shown to be less than 24 h old [4], so that their measurement is useful to estimate the platelet production by the bone marrow as it has been shown for human patients [5, 6]. Megakaryopoiesis can be estimated by evaluating a bone marrow aspirate or biopsy; however, taking a blood sample is much easier, less invasive and therefore the better choice for a continuous monitoring of the patient. Moreover, evaluation of bone marrow samples is subjective, time consuming and requires a highly skilled examiner. Measuring r-PLTs in whole blood using flow cytometry with Thiazol Orange is noninvasive and objective, but the method is both expensive and time-consuming. The Sysmex XE-2100 and the closely related Sysmex XT-2000iV are automated hematology analyzers designed for their use in human and animal specimens, respectively. Both analyzers measure platelets by impedance (PLT-I) and laser based methods (PLT-O). Due to its option of manual gating, the Sysmex analyzer XT-2000iV is capable of detecting immature r-PLTs by flow cytometry in the PLT-O channel, whereby a proprietary dye containing polymethine and oxazine is applied. The dye penetrates cell membranes and stains nucleic acids in reticulocytes and immature platelets. These cells are classified by flow cytometry based on their size (forward light scatter) and their fluorescence intensity (nucleic acid content) [7]. Since 2009, the detection of r-PLTs in dogs with the Sysmex XT-2000iV automated hematology analyzer has been evaluated and a reference interval has been established (*n* = 40 dogs [8] and 86 dogs respectively [9]).

Similar to human medicine [10], a large reference population of > 120 reference individuals has been recommended also in veterinary medicine to reliably establish reference intervals (ASVCP recommendations) [11]. To the authors’ knowledge, reference intervals based on such a large reference population have not been established for r-PLTs and platelet indices for the Sysmex XT-2000iV (thereafter XT-2000iV) analyzer before. Moreover, the impact of potentially interfering cell populations such as small erythrocytes and reticulocytes on r-PLT measurements performed with the manual gates published previously is largely unknown.

Several platelet indices such as mean platelet volume (MPV), platelet distribution width (PDW), platelet large cell ratio (P-LCR) and plateletcrit (PCT) are provided automatically by the XT-2000iV with each measurement. The diagnostic use of platelet indices reflecting platelet size as marker for juvenile platelets has been controversially discussed [12–17]. We previously demonstrated that there was no difference between platelet indices assessed with the XT-2000iV in thrombocytopenic dogs and controls despite a significantly higher percentage of r-PLTs observed in the diseased dogs [8]. However, the number of dogs evaluated previously was comparatively small (*n* = 40 control dogs, *n* = 8 thrombocytopenic dogs) so that a potential association between mean platelet volume (MPV) and the number of r-PLTs might have been missed. In contrast, the MPV was significantly higher in dogs with immune mediated thrombocytopenia than in healthy control dogs [18].

The aims of our study were 1) to compare an optimized gate with the two previously published gating methods [8, 9], 2) to assess the impact of potentially interfering cellular populations on r-PLT measurements, 3) to optimize the gates for measurement of r-PLTs with the XT-2000iV to eliminate possible interferences, and 4) to establish reference intervals for r-PLTs measured with the own and the previously published gating methods as well as for platelet indices in a reference population of > 120 dogs as recommended by the ASVCP [11].

## Methods

Data of 362 canine complete blood cell counts (CBCs) including 14 follow-up samples was analyzed retrospectively. Follow-up samples are defined as blood samples, which have been taken from the same diseased dog but at different dates. The blood samples have been collected between July 2006 and April 2007 in the department of veterinary clinical sciences, Giessen, Germany and between June 2009 and February 2010 in the department of Global Non-Clinical Safety, Merck Serono, Darmstadt, Germany. Overall, 169/362 samples were obtained from diseased dogs and 193/362 samples were taken from healthy dogs respectively.

### Analysis of r-PLTs and PLT parameters

**Fig. 1:**
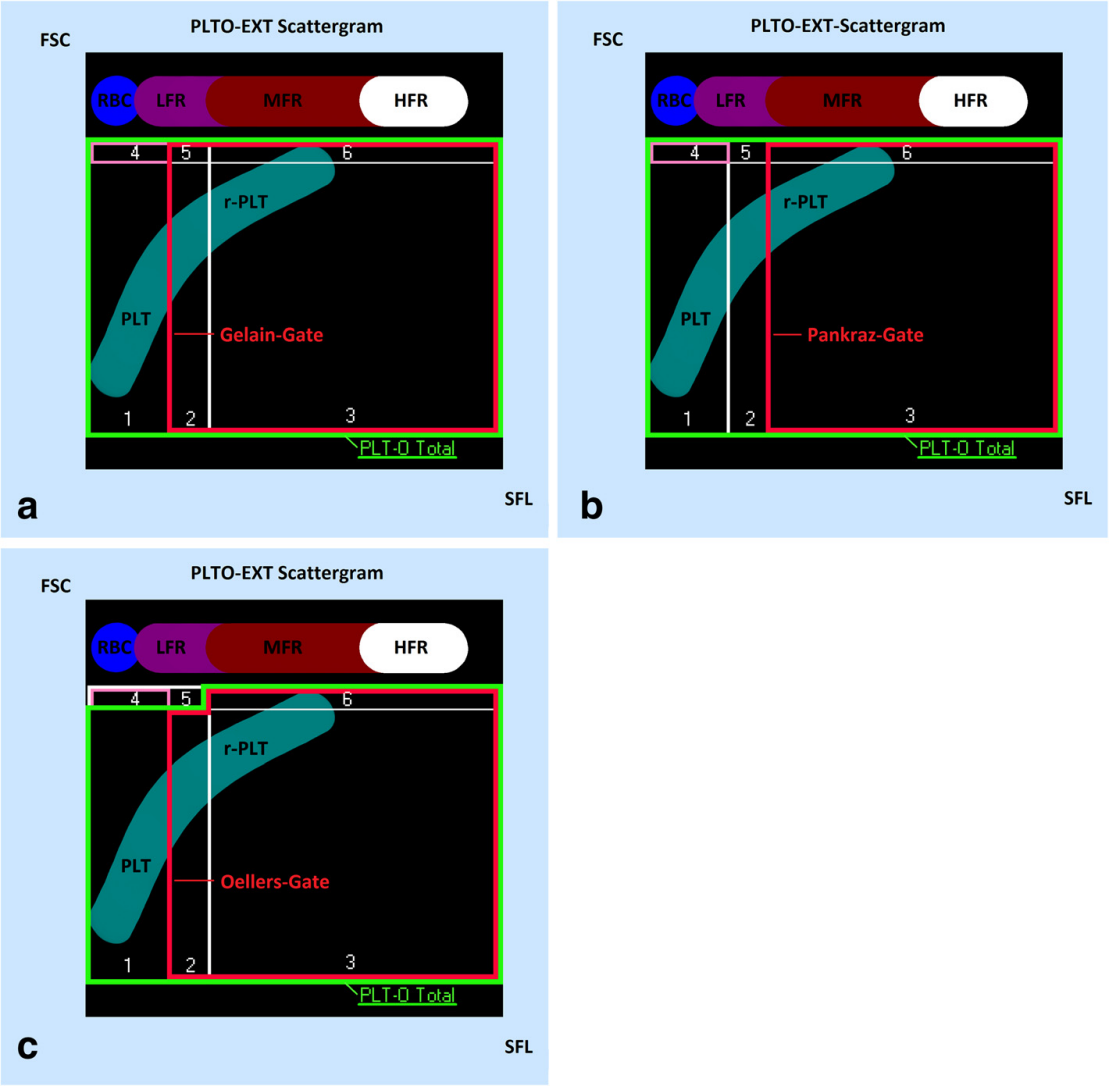
Schematic diagram showing the applied gates: “Gelain-gate” (**a**), “Pankraz-gate” (**b**) and “Oellers-gate” (**c**). Abbreviations: FSC, forward scatter; SFL, fluorescence intensity; RBC, erythrocytes; LFR, MFR, HFR, low, medium and high fluorescence ratio of reticulocytes; PLT, platelets; r-PLT, reticulated platelets; PLT-O total, green gate with all optically measured platelets; red gate, gate with r-PLTs; pink gate 4, gate with frequent interferences in the “Gelain-gate” and “Pankraz-gate”; white gate, can be ignored, not part of respective measurements

**Fig. 2:**
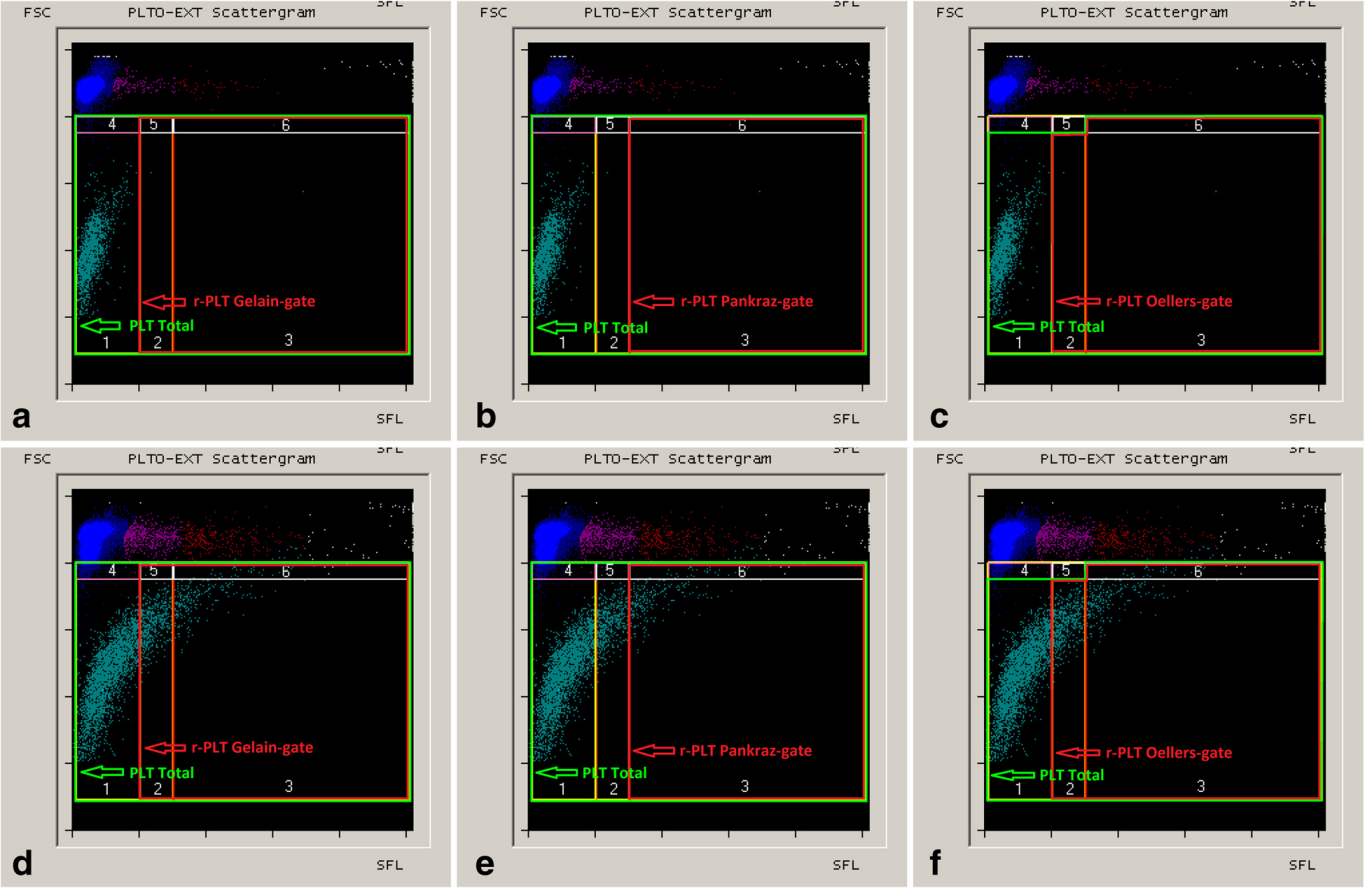
Scattergrams of the PLT-O channel of the Sysmex XT-2000iV analyzing whole blood samples of a healthy dog (**a**-**c**) and a dog with increased platelet and r-PLT count (**d**-**f**). The applied gates are shown schematically. PLT Total detects all platelets, (**a**, **d**) r-PLT “Gelain – gate” based on publication of Gelain et al., (**b**, **e**) r-PLT “Pankraz-gate” based on the publication of Pankraz et al., (**c**, **f**) r-PLT “Oellers-gate” shows the novel area for counting r-PLTs. Abbreviations: FSC, forward scatter; SFL, fluorescence intensity

Measurement was performed with ethylenediamine tetra-acetic acid (EDTA) anticoagulated blood samples using the XT-2000iV hematology analyzer and the veterinary software version 00–08 and 00–10. The retrospective analysis and gating was performed using the veterinary software version 00–11. To detect the number of r-PLTs in the blood samples, we created one gate to quantify the whole platelet population (Fig. 1 and 2, PLT-O total) and another gate located inside the first gate (Figs. 1 and 2, red gate) containing the r-PLTs. A modified version of the “Gelain-gate” was created to optimize the separation between mature and r-PLTs as well as to exclude erythrocytes and reticulocytes. This modified gate (“Oellers-gate”, Fig. 1c) was compared with the gates published by Pankraz et al. and Gelain et al. (“Pankraz-gate” Fig. 1b and Fig. 2b and e, “Gelain-gate” Fig. 1a and Fig. 2a and d). The risk of small erythrocytes and/or reticulocytes causing clinically important interferences and thus a false r-PLT count was given when gate 4 (Fig. 1) contained more than 5 % of the sum of dots measured in gates 1 and 4. The suspicion of potential interferences of small erythrocytes/reticulocytes with the r-PLT count was confirmed by visual control of the matching scattergram.

Retrospective analysis of each CBC included the following variables: platelet count by optical (PLT-O) and impedance methods (PLT-I), MPV, platelet distribution width (PDW), platelet large cell ratio (P-LCR), and plateletcrit (PCT).

The analyzer-specific canine reference intervals established previously for the XT-2000iV [19] were used as cut-off values to define anemia or regeneration of erythrocytes (i.e., anemia was diagnosed when the RBC count was < 5.1 × 10^12^/L and a reticulocyte count > 150.1 × 10^9^/L was considered regenerative).

### Reference population

The CBCs obtained from healthy dogs (*n* = 193, one sample per dog) included mainly samples taken from healthy Beagle dogs (153/193) obtained from one breeder (Marshall BioResources, USA). The Beagle dogs were kept under defined housing conditions: For 20 h a day, they were housed in groups of three to five dogs. Each dog spent the remaining four hours separated from the others in a standard kennel of 6.54 m^2^ and natural light. The Beagle dogs were fed once a day with 300 g of a commercially available pelleted dry food (Provimi Kliba AG, Kaiseraugust, Switzerland). Water was offered ad libitum. All Beagle dogs were dewormed and vaccinated against canine distemper, leptospirosis, canine parvovirus, parainfluenza, Bordetella, canine adenovirus 2, and rabies.

The other 40/193 healthy dogs kept as pet dogs were of various breeds including mixed-breed dogs (*n* = 17), Border Collies (*n* = 6), Great Danes (*n* = 3), West Highland White Terrier (*n* = 2), and one Beagle dog, Berger Blanc Suisse, Cocker Spaniel, Dalmatian, German Shepherd, Golden Retriever, Groenendael, Jack Russel Terrier, Labrador Retriever, Old German Shepherd Dog, Rough Collie, and Small Munsterlander each.

### Statistical analysis

Data was analyzed using Microsoft Excel®, Reference Value Adviser© version 2.1 for Microsoft Excel®, Analyse-it® version 2.04 for Microsoft Excel®, and GraphPad Prism® version 6. Data distribution was assessed visually with a histogram.

Spearman’s rank correlation, Passing-Bablok-Analysis, and Bland-Altman-Analysis were performed to determine correlation and bias between r-PLTs measurements obtained with the various gates. A Kruskal-Wallis-Test was used to assess the impact of the gating method on r-PLT results, whereby the *P* values were adapted for multiple comparisons. Level of significance was set at *P* < 0.05.

Intra-assay repeatability was assessed by 25 repeated measurements of one blood sample. For platelet indices, the XT-2000iV did not report results in 18/25 measurements so that calculation of intra-assay CV was based on 7 results.

Reference intervals for r-PLTs and platelet indices were generated for all healthy dogs independent of the breed (*n* = 193), and also for the subgroups “Beagles” (*n* = 153) and “non-Beagles” (*n* = 40). As recommended by the American Society of Veterinary Clinical Pathology (ASVCP) [11], the statistical method used for calculation of the respective reference intervals was chosen based on the number of dogs included in the respective reference population and data distribution. The whole group of healthy dogs (*n* = 193) and the group of Beagle dogs (*n* = 153) included ≥ 120 individuals, so that the nonparametric method was chosen to calculate reference intervals [11]. The group of pet dogs contained 40 (r-PLT, PLT-O, PLT-I) and 38 (MPV, PDW, P-LCR, PCT) dogs, respectively. When the number of samples was 40 ≤ × ≤ 120, the robust method was chosen for symmetrical (Gaussian) data distribution [11]. In case of non-Gaussian distribution, a Box-Cox transformation was performed prior to calculation of reference intervals. Depending on data distribution after Box-Cox transformation, a the parametric method was chosen for data with Gaussian distribution, while a nonparametric method was used in case of non-Gaussian distribution. As the XT-2000iV did not report MPV, PDW, P-LCR and PCT for 3/193 healthy dogs, data of merely 38/40 samples was available in the group of “non-Beagles” for these variables. As recommended previously for reference populations of < 40 individuals [11], the parametric method and robust method were used in case of Gaussian and non-Gaussian distribution, respectively. For all reference intervals, the 90 % confidence interval (CI) of the upper and lower ends of the reference limits was calculated using bootstrap methods.

## Results

The group of healthy Beagles included dogs with an age ranging from 10 to 14 months and equally distributed sex (77/153 intact males and 76/153 intact females).

In the group of healthy “non-Beagles”, 40 dogs (11/40 intact males, 7/40 castrated males, 5/40 intact females, as well as 17/40 castrated females) with a median age of two years (range: 4 months to 15 years) were enrolled.

In contrast, the diseased dogs (169/362) had a median age of seven years ranging between 3 months and 16 years. Overall, 60/169 intact males, 25/169 castrated males, 42/169 intact females as well as 42/169 castrated females were included.

In all 362 CBCs obtained from healthy and diseased dogs, the PLT count ranged between 3 × 10^9^/L and 871 × 10^9^/L. The r-PLT count as measured with the “Oellers-gate” ranged between 0.12 and 57.34 % in the whole group of 362 healthy and diseased dogs and between 0.29 and 57.34 % when regarding the group of diseased dogs alone (169/362).

The categories of underlying diseases are shown in Table 1. Overall, 41/169 samples were anemic with RBC counts < 5.1 × 10^12^ /L, whereby 19/41 anemic specimen showed regeneration of erythrocytes at the time of sampling with reticulocyte counts (RET) > 150.1 × 10^9^/L.

**Table 1:** Classification in categories of underlying etiology in diseased dogs

Category of disease	Absolute number (percentage)
Inflammatory diseases	31 (19.87 %)
Cardiovascular diseases	20 (12.82 %)
Neoplasia	18 (11.54 %)
Diseases of the musculoskeletal system	17 (10.90 %)
Monitoring after surgery	8 (5.13 %)
Immune-mediated diseases	
- Immune mediated anemia (*n* = 4/8) - Immune mediated thrombocytopenia (*n* = 3/8) - Evans syndrome (*n* = 1/8)	8 (5.13 %)
Portosystemic shunt	6 (3.85 %)
Diseases of the central nervous system	4 (2.56 %)
Non-regenerative anemia	4 (2.56 %)
DIC	2 (1.28 %)
Endocrinologic disease	2 (1.28 %)
Thrombosis	2 (1.28 %)
Regenerative anemia (unknown etiology)	1 (0.64 %)
Hemolytic anemia (unknown etiology)	1 (0.64 %)
Other diseases	32

*Abbreviations*: *DIC* disseminated intravascular coagulation

All three evaluated gates of the XT-2000iV showed good correlation as shown in Table 2. The best correlation was found between the “Gelain-gate” and the “Oellers-gate”. There was a small bias between the results obtained with the “Gelain-gate” and the “Oellers-gate”; however, the difference was not statistically significant (Table 2). In contrast, the “Pankraz-gate” differed significantly from the two other gates and there was a bias of approximately 2 %. The corresponding median value and range are shown in Table 3.

**Table 2:** Correlation and differences between the different gates for canine r-PLT measurement (*n* = 362) of the Sysmex XT-2000iV

Variable	r_s_	Slope (95 % CI)	Intercept (95 % CI)	Bias (95 % limits of agreement)	Adjusted *P*-value
r-PLT “Gelain-gate”(%) vs. r-PLT “Oellers-gate”(%)	1.00	1.01 (1.01–1.01)	0.00 (0.00–0.00)	0.1 (−1.3–1.6)	ns
r-PLT “Pankraz-gate”(%) vs. r-PLT “Gelain-gate”(%)	0.89	2.83 (2.70–2.99)	0.18 (0.12–0.23)	2.0 (−4.3–8.3)	<0.0001
r-PLT “Pankraz-gate”(%) vs. r-PLT “Oellers-gate”(%)	0.88	2.92 (2.78–3.11)	0.17 (0.10–0.22)	2.1 (−4.9–9.2)	<0.0001

*Abbreviations*: *CI* confidence interval, *ns* not significant, *vs*. versus

**Table 3:** Median, minimum and maximum r-PLT counts of the analyzed group of healthy and diseased dogs (*n* = 362)

Variable	Median	Minimum	Maximum
r-PLT “Gelain-gate”(%)	1.33	0.11	54.89
r-PLT “Pankraz-gate”(%)	0.40	0.0	35.02
r-PLT “Oellers-gate”(%)	1.33	0.12	57.34

**Fig. 3:**
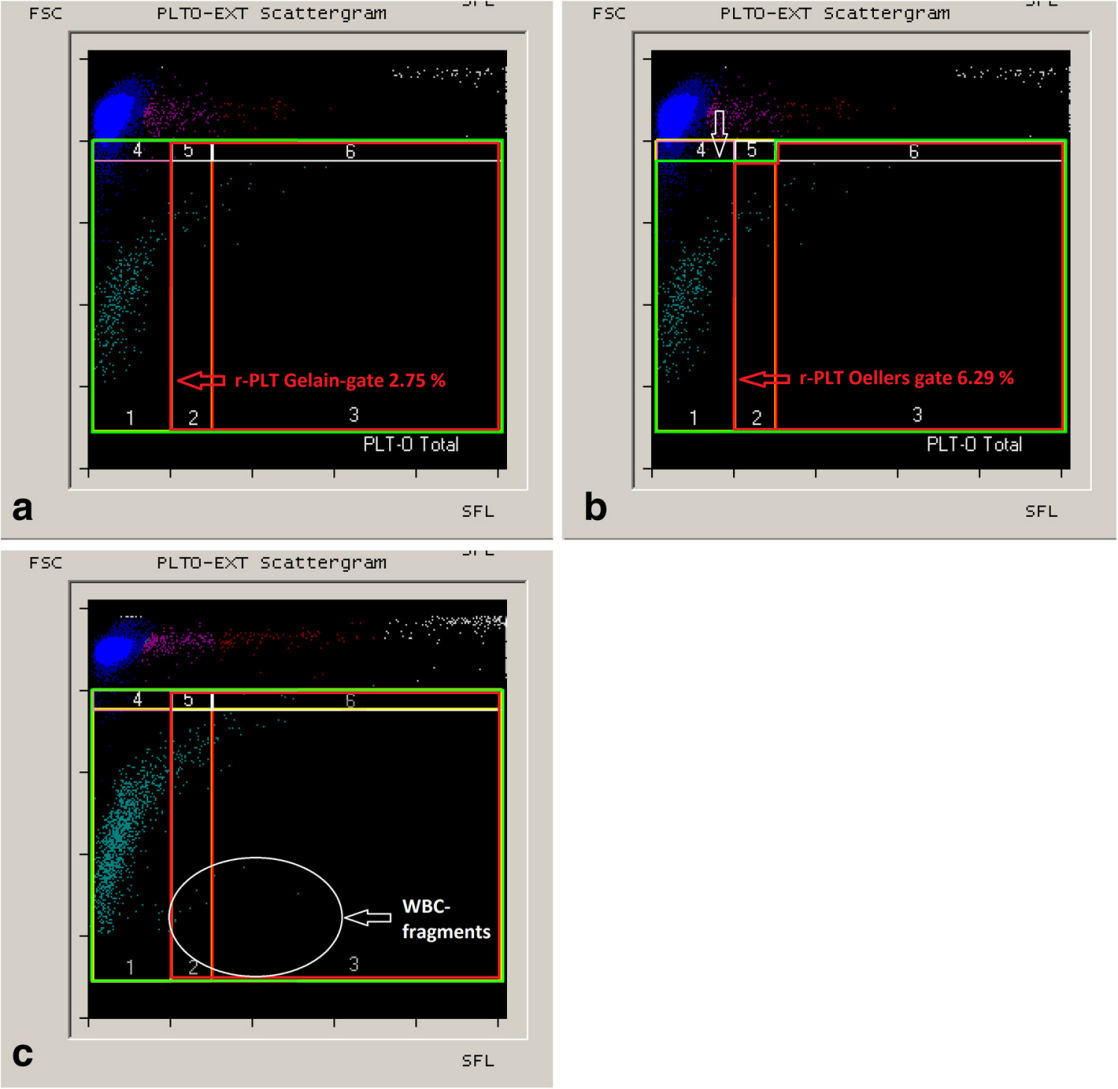
Samples of diseased dogs with suspected interferences. (**a**-**b**) Differences in the r-PLT percentage between the “Gelain-gate” (**a**) and the “Oellers-gate” (**b**) in a sample with suspected interferences of erythrocytes and reticulocytes. The white arrow shows the difference between the previously used gates and the novel “Oellers-gate”. (**c**) A sample with dots suspicious for WBC fragments appearing in the PLT-O channel and the gate for r-PLTs. Abbreviations: FSC, forward scatter; SFL, fluorescence intensity

In 30/362 samples, a number of dots exceeding 5 % of the sum of dots in gates 1 and 4 was found in gate 4 (Fig. 2d and e). In all cases, the visual control of the matching scattergrams showed an interference of small erythrocytes and/or reticulocytes. Gate 4 was included in both the “Gelain-gate” and the “Pankraz-gate” but excluded in the “Oellers-gate”. In Fig. 3a and b, one of 30 samples with clinically important interferences and their impact on the r-PLT measurement performed with the different gating methods is shown.

Furthermore, in 6/362 samples, visual control of the scattergrams revealed a small number of dots other than r-PLTs displayed in all evaluated r-PLT gates (Fig. 3c). These dots were positioned underneath the r-PLT population and were consistent with white cell fragments described in humans. However, in the present study, they made up a negligible number of dots.

For measurement of r-PLTs in all three gates, intra-assay CVs > 22.0 % were obtained. The highest CV of 41.1 % was seen for the “Pankraz-gate”. CVs for PLT-O, PDW and PCT were 5.6, 6.8 and 4.5 % and thus markedly lower than for r-PLT measurement. For the variables MPV, P-LCR and PLT-I, CVs < 3 % were obtained (Table 4). However, in 18/25 measurements, results of the platelet indices MPV, PDW, P-LCR and PCT were not reported by the analyzer due to an error in (data) analysis, so that calculation of the CV was based on only 7 repeated measurements.

**Table 4:** Intra-assay repeatability of r-PLT and platelet indices using the Sysmex XT-2000iV

Variable	CV (%)	Mean ± SD
r-PLT “Gelain-gate”(%)	22.02	6.98 ± 1.54
r-PLT “Pankraz-gate”(%)	41.14	1.81 ± 0.75
r-PLT “Oellers-gate”(%)	22.04	7.02 ± 1.55
r-PLT “Gelain-gate”(× 10^9^/L)	21.36	7.11 ± 1.52
r-PLT “Pankraz-gate”(× 10^9^/L)	40.10	1.84 ± 0.74
r-PLT “Oellers-gate”(× 10^9^/L)	21.38	7.1 ± 1.52
PLT-I (× 10^9^/L)	2.98	128.32 ± 3.83
PLT-O (× 10^9^/L)	5.56	121.2 ± 6.73
MPV (fL)	1.86	13.2 ± 0.24
PDW (fL)	6.75	17.77 ± 1.20
P-LCR (%)	2.72	47.7 ± 1.30
PCT (%)	4.52	0.17 ± 0.01

r-PLT, PLT-I, PLT-O: *n* = 25 repeated measurements

MPV, PDW, P-LCR, PCT: *n* = 7 repeated measurements

*Abbreviations*: *CV* coefficient of variation, *SD* standard deviation, *PLT-I* platelet count with impedance, *PLT-O* platelet count in optical fluorescence analysis, *MPV* mean platelet volume, *PDW* calculated distribution width of platelets, *P-LCR* platelet-large cell ratio, *PCT* plateletcrit, *L* liter, *fL* femtoliter

**Fig. 4:**
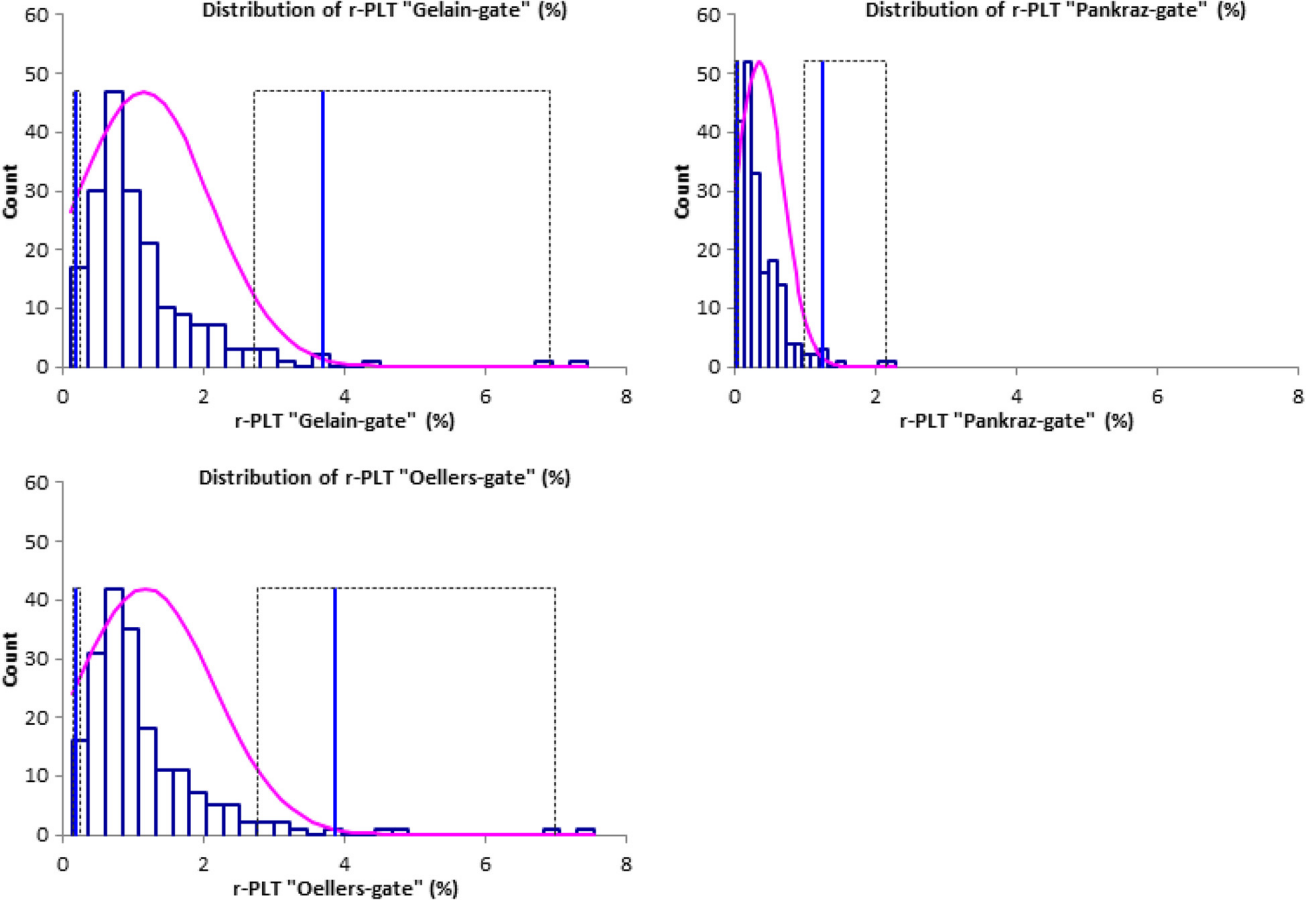
Reference interval and data distributions for r-PLTs of 193 healthy dogs measured with the Sysmex XT-2000iV and three different gating methods. Blue bars show the observed data distribution, the purple curve represents Gaussian fit. The blue vertical lines are consistent with the lower and upper limits of the reference interval; the dotted lines indicate the 90 % confidence interval. Abbreviations: r-PLT “Gelain-gate”: gate based on the publication of Gelain et al.; r-PLT “Pankraz-gate”: gate based on the publication of Pankraz et al., r-PLT “Oellers-gate”: optimized gate for measurement of r-PLTs

The reference intervals for r-PLTs obtained with the different gates are shown in Table 5. The corresponding data distribution is demonstrated in Fig. 4. Table 6 shows the reference ranges for platelet indices. Reference intervals obtained for all dogs irrespective of the breed, differed evidently from those calculated exclusively for the population of “Beagles” or “non-Beagles”. Overall, reference intervals established solely for the Beagle population showed the smallest range.

**Table 5:** Reference ranges for reticulated platelets (r-PLT) in healthy dogs independent of the breed as well as in Beagles and non-Beagles using the Sysmex XT-2000iV

Variable	All dogs (*n* = 193)			Beagles (*n* = 153)			Non-Beagles (*n* = 40)		
	2.5th Percentile (90 % CI)	97.5th Percentile (90 % CI)	Method	2.5th Percentile (90 % CI)	97.5th Percentile (90 % CI)	Method	2.5th Percentile (90 % CI)	97.5th Percentile (90 % CI)	Method
r-PLT “Gelain-gate”(%)	0.2 (0.1–0.3)	3.7 (2.7–6.9)	Nonparametric	0.2 (0.1–0.3)	2.4 (2.1–4.5)	Nonparametric	0.2 (0.1–0.3)	6.8 (4.8–9.3)	Robust, Box-Cox-Transformation
r-PLT “Pankraz-gate”(%)	0.0 (0.0–0.0)	1.2 (1.0–2.2)	Nonparametric	0.0 (0.0–0.0)	1.0 (0.7–1.3)	Nonparametric	0.0 (0.0–0.0)	2.2 (1.6–2.8)	Robust, Box-Cox-Transformation
r-PLT “Oellers-gate”(%)	0.2 (0.1–0.3)	3.9 (2.8–7.0)	Nonparametric	0.2 (0.1–0.3)	2.4 (2.1–4.5)	Nonparametric	0.2 (0.1–0.3)	7.1 (5.0–9.7)	Robust, Box-Cox-Transformation
r-PLT “Gelain-gate”(× 10^9^/L)	0.4 (0.3–0.6)	5.7 (4.9–8.7)	Nonparametric	0.4 (0.3–0.5)	4.2 (3.7–6.5)	Nonparametric	0.3 (0.1–0.4)	6.4 (5.0–8.0)	Robust, Box-Cox-Transformation
r-PLT “Pankraz-gate”(× 10^9^/L)	0.1 (0.0–0.1)	2.1 (1.8–2.9)	Nonparametric	0.1 (0.0–0.1)	1.7 (1.4–2.2)	Nonparametric	0.0 (0.0–0.1)	3.2 (2.7–3.2)	Nonparametric
r-PLT “Oellers-gate”(× 10^9^/L)	0.4 (0.3–0.6)	5.6 (4.9–8.7)	Nonparametric	0.4 (0.3–0.5)	4.2 (3.6–6.4)	Nonparametric	0.5 (0.3–0.7)	10.0 (7.5–12.6)	Robust, Box-Cox-Transformation

*Abbreviations*: *CI* confidence interval, for remainder abbreviations: see Table 4

**Table 6:** Reference ranges for platelet indices in healthy dogs independent of the breed as well as in Beagles and non-Beagles using the Sysmex XT-2000iV

Variable	All dogs (*n* = 190–193)			Beagles (*n* = 152–153)			Non-Beagles (*n* = 38–40)		
	2.5th Percentile (90 % CI)	97.5th Percentile (90 % CI)	Method	2.5th Percentile (90 % CI)	97.5th Percentile (90 % CI)	Method	2.5th Percentile (90 % CI)	97.5th Percentile (90 % CI)	Method
PLT-I (× 10^9^/L)	161.8 (149.0–175.0)	425.0 (381.0–438.0)	Non-parametric	176.7 (155.0–194.0)	406.9 (377.0–452.0)	Non-parametric	125.6 (115.5–143.7)	452.0 (405.3–501.8)	Robust, Box-Cox-Trans-formation
PLT-O (× 10^9^/L)	150.9 (141.0–169.0)	397.5 (355.0–416.0)	Non-parametric	172.8 (161.0–184.0)	377.1 (351.0–416.0)	Non-parametric	121.6 (110.6–137.1)	418.9 (375.2–467.7)	Robust, Box-Cox-Trans-formation
MPV (fL)	8.6 (8.3–8.9)	12.5 (12.1–12.8)	Non-parametric	8.8 (8.6–8.9)	12.3 (12.1–13.4)	Non-parametric	7.9 (7.4–8.4)	12.8 (12.3–13.4)	Robust
PDW (fL)	8.8 (8.4–9.2)	16.6 (14.7–18.0)	Non-parametric	9.0 (8.7–9.3)	15.4 (14.5–18.0)	Non-parametric	7.9 (7.5–8.6)	18.3 (16.4–20.5)	Robust, Box-Cox-Transformation
P-LCR (%)	14.1 (12.1–15.9)	43.0 (41.3–44.7)	Non-parametric	15.5 (14.1–16.8)	43.1 (41.3–48.2)	Non-parametric	9.1 (5.3–13.1)	45.8 (41.5–49.8)	Parametric
PCT (%)	0.2 (0.2–0.2)	0.4 (0.4–0.4)	Non-parametric	0.2 (0.2–0.2)	0.4 (0.4–0.5)	Non-parametric	0.2 (0.1–0.2)	0.4 (0.4–0.5)	Robust, Box-Cox-Transformation

*Abbreviations*: *CI* confidence interval; for remainder abbreviations: see Table 4

In 3/193 healthy dogs (1/153 Beagle and 2/40 non-Beagle), platelet indices were not reported by the analyzer due to an error in (data) analysis so that these three samples could not be included in the calculation of the respective reference intervals.

Furthermore, the analyzer did not report a result for the platelet indices in samples with r-PLT percentages exceeding 11.61 % (19/362) for the “Gelain-gate” and 11.66 % (21/362) for the “Oellers-gate”, respectively. In samples without reported PLT indices, the platelet histogram was characterized by a relatively indistinctive separation (“valley”) between erythrocytes and platelets so that a reliable differentiation between the cellular populations could not be ensured by the analyzer’s software.

## Discussion

Overall, the retrospective analysis of a large number of dog CBCs enabled us to optimize the already existing r-PLT gates by excluding interferences of reticulocytes and small erythrocytes with the r-PLT measurement.

The significant difference between the “Pankraz-gate” and the two other gates is presumably a result of its smaller size. Compared to the other gates, r-PLTs need to contain more RNA to be detected in the “Pankraz-gate”. Consequently, the corresponding reference ranges obtained for the “Pankraz-gate” are much narrower than for the other gates. The difference between the “Gelain-gate” and “Oellers-gate” was solely based on samples where erythrocytes and/or reticulocytes interfered with the measurement of platelets and r-PLTs. Using the “Gelain-gate”, these interfering erythrocytes and/or reticulocytes were incorrectly counted as mature platelets, consequently resulting in a false high total platelet count obtained with the manual gate and thus a false low percentage of r-PLTs. This incorrect cell count is only a problem of the manual gating technique; however, the original automated PLT count could not be used for the purposes of r-PLT measurement. In contrast, the effect of interferences, i.e., false low r-PLT counts, is minimized by the novel “Oellers-gate” by excluding the critical group of cellular populations (Fig. 3a and b).

Another reason for visual examination of the scattergrams is the occurrence of another type of interfering cells within the r-PLT-gate, which were rarely observed in the present study (Fig. 3c) but have not been described previously for dogs. In human medicine, similar interferences have been reported in patients receiving chemotherapy and were considered to be white cell fragments [20]. In our study, the definite origin and etiology of the interferences could not be evaluated.

The r-PLT reference intervals obtained in this study are consistent with the reference intervals published previously for the XT-2000iV [8, 9]. Reference intervals established here for the “Gelain-gate” (0.2–3.7 %; 0.4–5.7 × 10^9^/L) and the “Oellers-gate” (0.2–3.9 %; 0.4–5.6 × 10^9^/L) in 193 dogs are comparable with results published previously by Gelain et al. [9] (1.4 ± 0.7 %; 4 ± 2.5 × 10^9^/L, *n* = 86). Furthermore, our reference interval established for the “Pankraz-gate” (0.0–1.2 %) is in accordance to the reference range shown previously by Pankraz et al. for the XT-2000iV [8] (0.56 ± 0.82 %, *n* = 40).

Our results clearly showed that the reference intervals in Beagle dogs are much smaller than in non-Beagle dogs, which is most likely because of the lower genetic variation, the similar age and the identical housing conditions.

Moreover, the reference intervals published by Smith et al. for flow cytometry with Thiazol Orange and CD61 [21] are also comparable (1.9 ± 1.2 %; 5.2 ± 3.4 × 10^9^/L, *n* = 36) with our results. However, when regarding other reference intervals for r-PLTs determined flow-cytometrically with the Thiazol Orange method, there is a large discrepancy between results reported in the literature ranging from 3.4 ± 2.0 % (*n* = 8 dogs) [22] to 9.3 ± 2.7 % (*n* = 20 dogs) [23] and the respective reference intervals are generally higher than those obtained in our study. Overall, it is obvious that reference intervals for flow cytometry differ clearly between different studies, whereas reference intervals for the XT-2000iV using similar gates showed good accordance. A possible cause could be different methodology, i.e., variations in staining protocols or gating of r-PLTs in laboratories using flow cytometry [24].

Furthermore, our reference intervals for platelet indices (*n* = 190–193, depending on the variable) showed good accordance with reference ranges published before for the XT-2000iV (*n* = 160–182, *n* = 126–132, depending on the variable [19, 25]).

However, the study is limited by its retrospective nature so that a comparison with a reference method other than the previously published gates was not performed. For interpretation of the reference intervals, it has to be considered that the group of non-Beagle dogs is also smaller than recommended by the ASVCP [11] and reference intervals have to be therefore considered as a rough estimate.

Despite the optimization of the gating method described here, interferences could not be totally avoided. Only flow cytometric assessment of specifically antibody-labeled platelets would allow a definite distinction between platelets and other cellular populations. Another potential limitation of the gating method applied by us and the previous authors evaluating the r-PLT count measured by the Sysmex XT is that the gates are based on visual – and therefore subjective and potentially imprecise – assessment of the dot plots. Additionally, the position and size of the gates is fixed, so that in some samples, the shape of the gates did not entirely match the shape of the total platelet population. Thus, each measurement should be verified by visual examination of the scattergram. Ideally, algorithms would be generated to adapt the gate’s position to the properties of the platelet population.

Similar to results of previous studies using the Sysmex XT-2000iV, the platelet indices MPV, PDW, P-LCR and PCT [8, 19, 25] are frequently not reported by the analyzer. Because of the retrospective nature of this study, resampling was not possible so that the intra-assay repeatability could not be calculated for platelet indices, which is a limitation of this study. It should be noted that in samples with more than 11.61 % r-PLTs, as measured with the “Gelain-gate”, no platelet indices were given. Consequently, the platelet indices cannot be used to reflect presence of large numbers of young – presumably large - platelets and consequently a marked platelet regeneration as has been controversially discussed in previous studies [12, 16, 26, 27].

## Conclusions

In conclusion, we managed to optimize the previously published gates by excluding interferences from erythrocytes. However, visual assessment of the scattergrams still remains necessary. Future improvement might be achieved by the generation of algorithms automatically adapting the gate to the position of the platelet population. The use of platelet indices as a marker of PLT regeneration is questionable as their measurement is often not reliably possible especially in samples with evidence of larger numbers of immature PLTs.

The reference intervals obtained from a large population of Beagle dogs for r-PLTs and platelet indices might become particularly useful for experimental studies using Beagles.
